# A Novel Wild-Type *Saccharomyces cerevisiae* Strain TSH1 in Scaling-Up of Solid-State Fermentation of Ethanol from Sweet Sorghum Stalks

**DOI:** 10.1371/journal.pone.0094480

**Published:** 2014-04-15

**Authors:** Ran Du, Jianbin Yan, Quanzhou Feng, Peipei Li, Lei Zhang, Sandra Chang, Shizhong Li

**Affiliations:** 1 Institute of Nuclear and New Energy Technology, Tsinghua University, Beijing, China; 2 School of Life Sciences, Tsinghua University, Beijing, China; 3 Beijing Engineering Research Center for Biofuels, Beijing, China; University of Nottingham, United Kingdom

## Abstract

The rising demand for bioethanol, the most common alternative to petroleum-derived fuel used worldwide, has encouraged a feedstock shift to non-food crops to reduce the competition for resources between food and energy production. Sweet sorghum has become one of the most promising non-food energy crops because of its high output and strong adaptive ability. However, the means by which sweet sorghum stalks can be cost-effectively utilized for ethanol fermentation in large-scale industrial production and commercialization remains unclear. In this study, we identified a novel *Saccharomyces cerevisiae* strain, TSH1, from the soil in which sweet sorghum stalks were stored. This strain exhibited excellent ethanol fermentative capacity and ability to withstand stressful solid-state fermentation conditions. Furthermore, we gradually scaled up from a 500-mL flask to a 127-m^3^ rotary-drum fermenter and eventually constructed a 550-m^3^ rotary-drum fermentation system to establish an efficient industrial fermentation platform based on TSH1. The batch fermentations were completed in less than 20 hours, with up to 96 tons of crushed sweet sorghum stalks in the 550-m^3^ fermenter reaching 88% of relative theoretical ethanol yield (RTEY). These results collectively demonstrate that ethanol solid-state fermentation technology can be a highly efficient and low-cost solution for utilizing sweet sorghum, providing a feasible and economical means of developing non-food bioethanol.

## Introduction

The need for energy security, the state of the global petroleum supply, increased air pollution, and climate changes have demanded the production of sustainable and renewable biofuels [1], [2]. Bioethanol is currently the most widely used liquid biofuel and is used as both a fuel and a gasoline enhancer [3]. However, increasing bioethanol production is beginning to cause several problems. For example, the cultivation of crops for fuel is resulting in competition for cropland, and the establishment of large palm and sugarcane plantations is destroying native ecosystems [2], [4], [5]. The need to resolve the competition between food and fuel has sparked a strong interest in developing new biofuel crops [2].

Indeed, sweet sorghum (*Sorghum bicolor* (L.) Moench) has become one of the most promising crops for fuel ethanol production, as it produces grains with high starch content, stalks with high sucrose content, and leaves with a high lignocellulosic content. Additionally, sweet sorghum exhibits high photosynthetic efficiency, a short growth period (3–5 months), increased drought and saline-alkali resistance, low fertilization requirements, and a wide cultivation range [6], [7]. These characteristics suggest that sweet sorghum possesses a high potential for large-scale ethanol production and related comprehensive use, and this plant has been considered as a promising alternative feedstock for bioethanol production worldwide [8].

However, it remains unclear how sweet sorghum can be cost-effectively utilized for ethanol production, which is an urgent problem that needs to be resolved. The most common method is liquid-state fermentation of sweet sorghum juice obtained through pressing of the plant. Although this method is technically simple and mature, the loss of total sugar during the pressing procedure [9], low ethanol fermentation content, and large amount of wastewater from fermentation further increase production costs [10]–[12]. Therefore, solid-state fermentation of sweet sorghum is gaining more attention because of the higher sugar utilization and ethanol yield, lower energy expenditure and capital cost, and reduced water usage and wastewater output [13], [14], which are aspects that are favorable for the development and implementation of industrial production. Recent breakthroughs, including the on-line monitoring and control of the materials and the fermenter [15], [16] and mathematical modeling of the process [14], [16], [17], have mainly been achieved at the laboratory scale [10], [11], [18], [19]. However, difficulties in scaling up restrict the further development of solid-state fermentation because crushed sweet sorghum stalks have poor free water and heat transfer capabilities, which further affect the stability and uniformity of the conditions (such as temperature, moisture content, and pH) that are critical in solid-state fermentation [13]–[15]. Due to these difficulties, previous study showed that the relative theoretical ethanol yield (RTEY) reached to only 75% when scale enlarged to 127 L as reported [19], which was still far from the industrial requirements to scale and conversion.

To determine a cost-effectively method for bioethanol production by sweet sorghum stalks at industrial-scale solid-state fermentation, we began by isolating strains that would be best suited to those conditions from the soil on which sweet sorghum stalks were stored. We identified a *Saccharomyces cerevisiae* strain, TSH-SC-1 (abbreviated as TSH1), which showed significant advantages for use in solid-state fermentation, including tolerance to high temperatures and low moisture content. We subsequently designed a series of rotary-drum fermenters from 50-L to 127-m^3^ based on the fermentation characteristics of TSH1 and determined the stability of TSH1 in these large fermentations. The results showed that TSH1 had a high and stable ethanol production rate (11.1±0.39 g/kg/h) and RTEY (88±0.8%) in progressively scaled-up fermentation. Finally, a commercial demonstration system with a 550-m^3^ rotary-drum fermenter was designed and constructed, and we demonstrated that up to 96 tons of crushed sweet sorghum can be fermented within just 20 hours, with a RTEY reaching 88% for an energy input/output ratio of 1∶2.6. Cost accounting also showed that the ethanol cost per ton is competitive in market compared with corn and sugarcane ethanol. Taken together, these results suggest a possible solution for the cost-effective production of non-food bioethanol through the utilization of sweet sorghum.

## Materials and Methods

### Strain isolation

Soil samples collections were permitted by Mr. Weiyi Yao on private land (North 18.53 by East 109.47) belonged to Hainan Agriculture and Green Agriculture Co., LTD, which was used for the long-term storage of sweet sorghum in Hainan Province, China. A single strain was isolated based on dilution separation methods [20]. For performance comparisons, the isolated stains were inoculated with 20 g of crushed sweet sorghum stalks in a 100-mL solid-state fermentation vial at an inoculum size of 1×10^7^ cells per gram crushed sweet sorghum stalks (70% moisture content). To compare the growth rate according to the volume of gas produced, the vials were sealed with butyl rubber plugs, and a 50-mL syringe was inserted into the rubber plug of each bottle for gas collection. The samples were incubated at 30°C for 7 hours and subsequently collected following a previously described method [21]. The ethanol concentrations were measured using HPLC with an Aminex HPX-87H column (Bio-Rad, Hercules, CA, USA) [22]. The ethanol production rate is defined as the ethanol weight produced per kg dry stalk per hour (g/kg/h) [10] as follows: 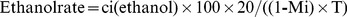
(1)where ci (ethanol) is ethanol content of the collected sample, Mi is moisture content after fermentation of the collected sample, T is fermentation time (hours).

TSH1 has been deposited in China General Microbiological Culture Collection Center (Accession number: CGMCC 1949).

### Species identification

A phylogenetic analysis was performed according to a previously described method [20]. A DNA fragment covering the internal transcribed spacer (ITS) region, 18S rDNA, and 26S rDNA D1/D2 domain was amplified with the primers ITS1 (5′-GTCGTAACAAGGTTTCCGTAGGTG-3′) and NL4 (5′-GGTCCGTGTTTCAAGACGG-3′) and sequenced. The sequence of TSH1 and the reference sequences were aligned with MEGA 4 and adjusted manually. Phylogenetic trees were constructed from the evolutionary distance data calculated from Kimura's two-parameter models using the neighbor-joining method [23]. Bootstrap analyses were performed on 1000 random replications [24].

For the analyses of morphological characteristics and budding, TSH1 was grown on YPD plates at 30°C for 12 hours. The cells were collected, diluted, and observed with a Nikon Ti-E Inverted Fluorescence Microscope (Nikon Instruments [Shanghai] CO., LTD, Shanghai, China) [25]. TSH1 was grown on McClary plates at 28°C for 7 days for ascospore detection [25], [26]. For ploidy identification, PCR was used to detect alleles of the mating type (MAT) locus (MATa or MATα) [27]. Two sets of primers were used: primer p1 (5'-AGTCACATCAAGATCGTTTATGG-3') and p2 (5'-GCACGGAATATGGGACTACTTCG-3') were used for MATa detection, and p2 and p3 (5'-ACTCCACTTCAAGTAAGAGTTTG-3') were used for MATα. *S. cerevisiae* Y294 was used as the haploid control [28], and *S. cerevisiae* BY4743 was used as the diploid control [29]. A biochemical identification kit (Bio-Kont 16C, Bio-Kont Technology Co., Ltd., China) was used for the determination of biochemical characteristics.

### Tolerance analysis

TSH1 was cultured in 250-mL flasks with 100 mL of yeast peptone dextrose (YPD) liquid medium. For the temperature tolerance analysis, the flasks were incubated at 30°C, 35°C, or 40°C, with a shaking speed of 150 rpm. For the product inhibition tolerance analysis, TSH1 was cultured in YPD liquid medium supplemented with different initial ethanol (0%, 5% and 6.5%), or different initial acetate (2 g/L, 3 g/L and 4 g/L), and cultured at 30°C with a shaking speed of 150 rpm. Hydrochloric acid was used to adjust the YPD medium to the indicated pH values to assess the acidic pH tolerance. Culture samples were collected at different time points, and the OD_600_ was measured until the stationary phase was reached. The growth curves were modeled using a logistic growth equation [30]. BY4743 was used as the control. The absolute growth rate (AGR) was calculated using the following equation: 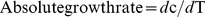
(2)where c is OD_600_ of the sample, T is incubation time (hours).

### Solid-state fermentation in flasks

1 kg of crushed sweet sorghum stalks and TSH1 were added to a shrunk rotary-drum fermenter, which was 0.25 m in length and 0.1 m in diameter, and then fully blended with TSH1 by rotating at 5 rpm in a 4°C incubator for 20 min. 500-mL flasks were used and each were loaded with 100-g of blended substrates. For the temperature tolerance analysis, the flasks were incubated at 30°C, 35°C, or 40°C for 12 hours; crushed sweet sorghum stalks with the indicated moisture contents including 40%, 50%, 60% and 70% were used for the moisture tolerance analysis. Samples were collected at the zero time point and at the end of fermentation, and the ethanol concentrations were measured by HPLC.

### Scaled-up fermentation at the laboratory scale

A 50-L fermenter (0.7 m in length, 0.3 m in diameter) was designed and constructed of stainless steel. A water jacket and insulation layer covered the surface of the fermenter for temperature regulation during fermentation. The temperature in the fermenter was monitored by eight temperature sensors at different locations. A screw-drive motor was used to rotate the fermenter, and a series of baffles fixed on the inside wall of the fermenter enhanced agitation during fermentation. Approximately 14 kg of crushed sweet sorghum was fully blended with 10% (v/w) TSH1 inoculum and loaded into the fermenter; fermentation was performed at 30°C for 12 hours with a rotary speed of 0.5 rpm. Samples were collected at the starting and end points of fermentation, and the ethanol concentration was determined by HPLC. The sugar concentration was determined using the 3,5-dinitrosalicylic acid (DNS) method [31]. The RTEY is defined as the ratio of the ethanol weight produced compared to the theoretical yield based on the consumed sugar (%) [10] as follows: 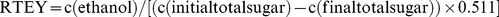
(3)where ci (ethanol) is ethanol content of the collected sample, ci (sugar) is total sugar content (initial sugar minus residual sugar after fermentation) of the collected sample as measured by the DNS method.

### Scaled-up fermentation at the pilot scale

A rotary-drum fermenter (approximately 5 m in length and 1 m in diameter) was designed and built based on the 50-L fermenter. However, a conveyor for feeding and discharging materials and a 5-degree inclination angle between the fermenter and base were included. Crushed sweet sorghum stalks were prepared by the evaporative cooling method [14], [15] to raise the substrate temperature to 28°C and the moisture content to approximately 66% prior to mixing with TSH1 inoculum during the loading process. Approximately 1 ton of mixture was loaded each time. Large fermenters with effective volumes of 40 m^3^ and 127 m^3^ were built based on the 5-m^3^ fermenter that can ferment approximately 4 tons and 22 tons, respectively, of crushed sweet sorghum stalks in each batch.

### Scaled-up fermentation at the commercial demonstration scale

The commercial demonstration scale rotary-drum fermenter was 55 m in length and 3.6 m in diameter, and the effective fermentation volume was 550-m^3^. The fermenter was driven by a screw drive with three supported gears in the front, middle, and back parts of the fermenter. A water jacket, insulation layer, and temperature sensors were used for temperature control and monitoring, and conveyors were used for feeding and discharging materials. A 5-degree angle was applied to transfer the substrate from the feeding inlet to the discharge outlet during rotation with the aid of optimized baffles [17] fixed on the inside wall of the fermenter. For each batch, approximately 96 tons of inoculated and crushed sweet sorghum stalks were loaded via the conveyors to the fermenter, and a slow rotation of the fermenter was used to enhance the mixing and transfer of materials inside the fermenter.

Residual sugar is defined as the percentage, by weight, of the total sugars (sucrose×1.05 + glucose + fructose) that remain unfermented in 1 g of dry stalk (%, (w/w)) [10] as follows: 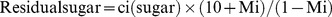
(4)where ci (total sugar) is total sugar concentration (sucrose×1.05 + glucose + fructose) of the collected sample as measured by the DNS method, Mi is moisture content of the collected sample, w (substrate) is substrate weight.

The ethanol yield is defined as the percentage, by weight, produced by 1 g of dry stalk (%, (w/w)) [10] as follows: 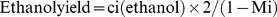
(5)where ci (ethanol) is ethanol concentration of the collected sample as measured by HPLC (g/L), Mi is moisture content of the collected sample.

## Results

### TSH1 can produce ethanol from sweet sorghum via solid-state fermentation

**Figure 1:**
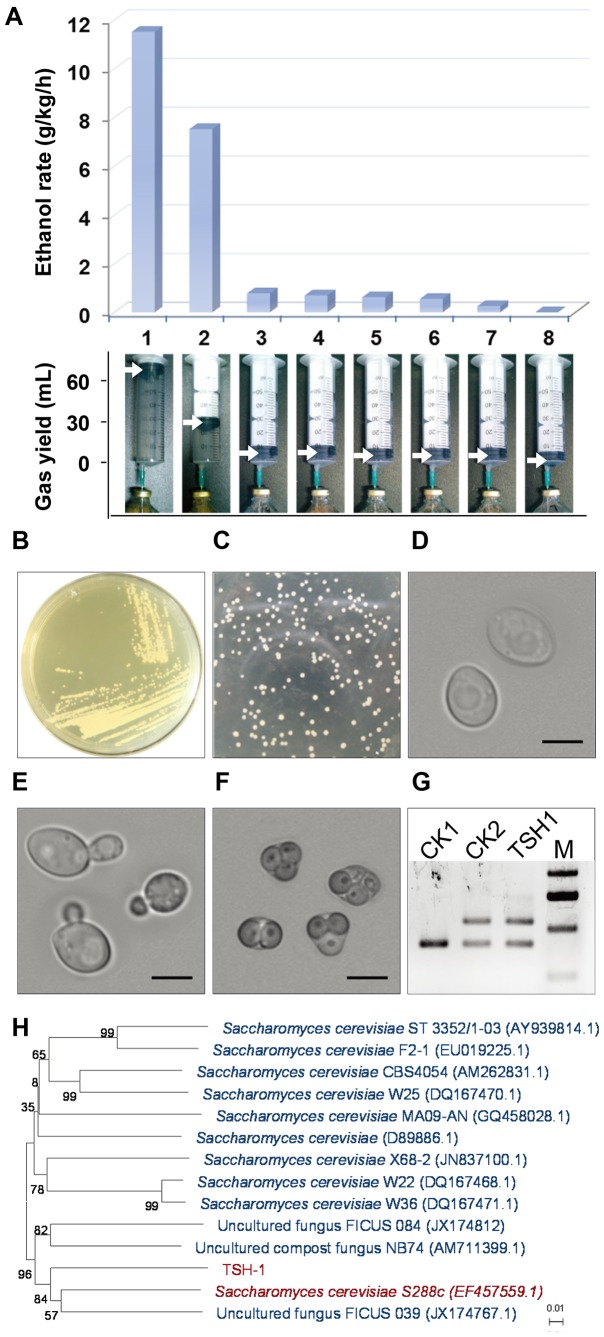
Isolation and identification of TSH1. (A). Ethanol fermentation capability of the screened strains. Eight strains isolated from soils in which sweet sorghum stocks were stored were cultured with crushed sweet sorghum as the substrate in 100-mL vials at 30°C for 7 hours. The ethanol content was determined by HPLC and is presented as the ethanol production rate in g/h per kg of crushed sweet sorghum (g/kg/h) in the top panel. Gas production analysis was performed with graduated syringes that were inserted into the rubber plugs of each vial before the start of the culture (bottom panel). The white arrows indicate the cumulative gas production during the 7-hour cultivation. The strain numbered “1” is TSH1, and 2–8 are the other strains screened. (B). Morphology of TSH1 grown on rich medium. TSH1 was cultured at 30°C for 12 hours on YPD plates. (C). Colony phenotype of TSH1 described in (B). (D). Cell morphology of TSH1. Images of vegetative cells grown on YPD medium were captured using a Nikon Ti-E Inverted Fluorescence Microscope. Magnification = 100×10. The bars represent 10 µm. (E). Cells of TSH1 in the budding (asexual reproduction) state. Cells formed buds on YPD medium after 12 hours at 30°C. The bars represent 10 µm. (F). TSH1 ascospore formation. TSH1 was grown on McClary medium for 7 days at 28°C. The bars represent 10 µm. (G). TSH1 ploidy analysis with the detection of MATa and MATα alleles. CK1 (*S. cerevisiae* Y294) was used as the haploid control and CK2 (*S. cerevisiae* BY4743) was used as the diploid control, and M represents the DNA ladder (from top to bottom: 750 bp, 500 bp, and 250 bp). (H). TSH1 is closely related to *S. cerevisiae* S288c. Phylogenetic tree reconstructed from the neighbor-joining analysis of the 18S rDNA gene and 26S rDNA sequence of TSH1. The bootstrap percentages over 50% (from 1000 bootstrap replicates) are shown. The reference sequences were from the species type strains retrieved from GenBank under the indicated accession numbers. The bars represent 0.01 substitutions per nucleotide position.

To compare the ethanol production via solid-state fermentation from sweet sorghum stalks, we established a mini solid-state fermentation system using vials filled with crushed sweet sorghum stalks and sealed with rubber plugs connected to syringes for monitoring gas production. The strains were inoculated into the vials and fermented at 30°C for 7 hours. After determining the ethanol concentrations using high performance liquid chromatography (HPLC), we found that one of the strains produced significantly more ethanol compared with the other strains, as shown in Figure 1A. We named this strain TSH-Sc-1 (TSH1). Consistent with its higher ethanol production capability, the strain exhibited the highest level of gas production of all the strains tested (Figure 1A), which suggested that it propagated well when using sweet sorghum stalks as the sole substrate.

Phenotypic observations (Figure 1B–F) showed that TSH1 exhibited morphological characteristics similar to those of *S. cerevisiae*, including a milky colony with a smooth surface, oval cells with diameters of 5–7 µm, and budding as well as spore reproduction. A gene sequence analysis of the internal transcribed spacer (ITS) region and 18S rDNA and 26S rDNA genes demonstrated that TSH1 is a novel strain of *S. cerevisiae* and is clustered in a branch with *S. cerevisiae* S288c (Figure 1H), a widely studied laboratory strain [32]. Yeast ploidy analysis [27], the detection of the mating type locus a (MATa) and mating type locus α (MATα) genes (Figure 1G) and the ability to reproduce via spores (Figure 1F) collectively further demonstrated that TSH1 is an a/α-type diploid *S. cerevisiae*.

To further investigate the physiological and biochemical characteristics of TSH1, we determined its assimilation and fermentation capabilities using different carbon and nitrogen sources. BY4743, the diploid type of S288c [29], was used as the control. Consistent with the close genetic relationship between TSH1 and BY4743, both strains exhibited the same physiological and biochemical characteristics, including the ability to assimilate glucose, fructose, maltose, sucrose, inositol, trehalose, lactose, galactose, dulcitol, and urea but not nitrate (Table 1).

**Table 1:** Assimilation and fermentation capability of different nutrient elements.

	TSH-1	BY4743
**Fermentation of:**		
Lactose	+	+
Sucrose	+	+
Glucose	+	+
Maltose	+	+
**Assimilation of:**		
Galactose	+	+
Sucrose	+	+
Glucose	+	+
Trehalose	+	+
Inositol	+	+
Dulcitol	+	+
Maltose	+	+
Urea	+	+
Nitrate	-	-

### TSH1 exhibited outstanding potentials in solid-state fermentation

The low heat and mass conduction ability of solid-state fermenters tends to create a non-uniform distribution of heat and products, leading to localized high temperatures and over-accumulation of product inhibitors (especially for ethanol and acetate) [16], [33]–[35], which requires strains to be adaptable to temperature fluctuations, products inhibitions.

**Figure 2:**
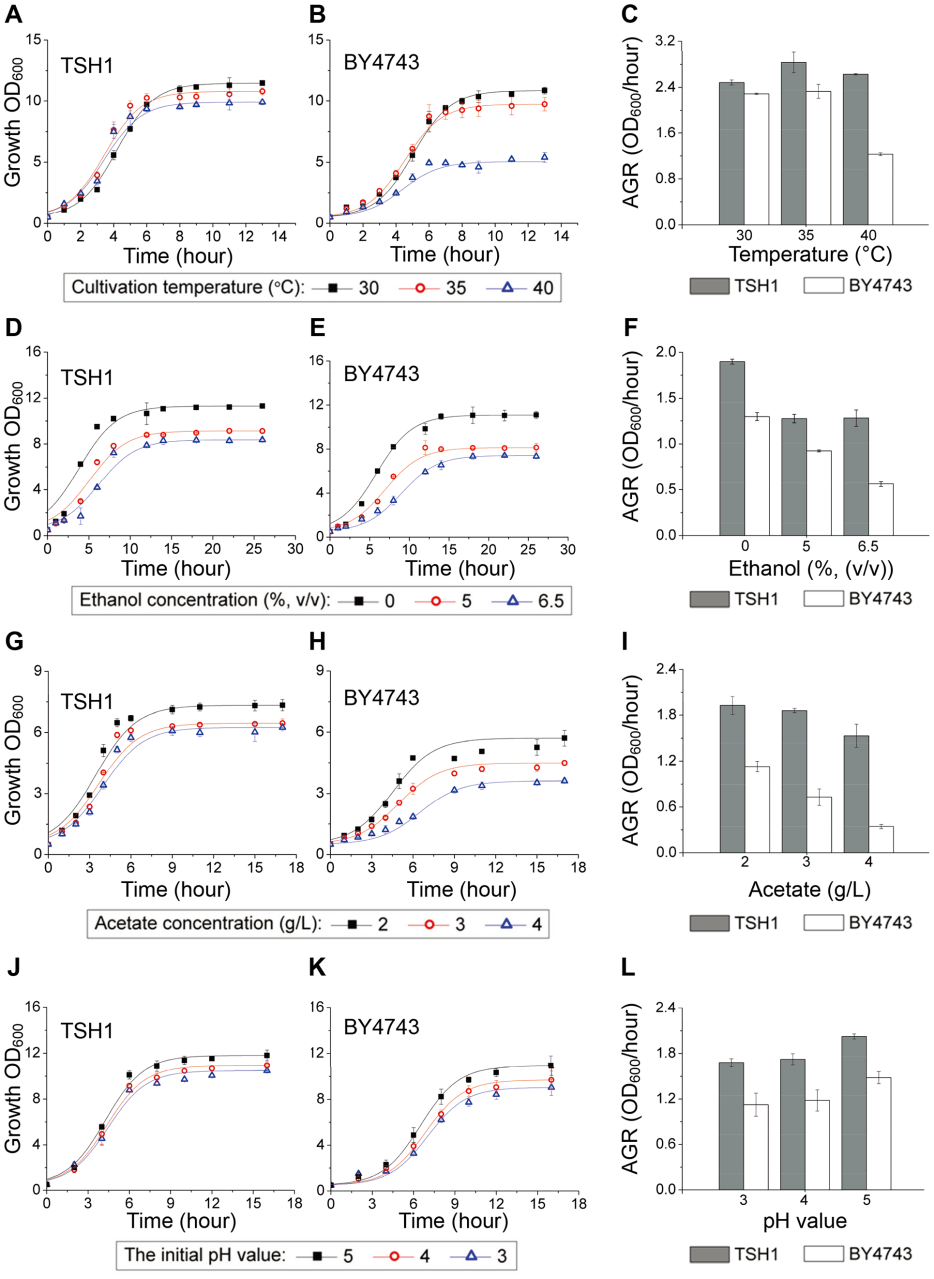
TSH1 exhibits good tolerance to different stress conditions. TSH1 was cultured under the indicated stress conditions until the growth curve reached the stationary phase. BY4743 served as the control strain. The growth curves of TSH1 (left panel) and BY4743 (middle panel) were constructed using OD_600_ values and fitted by a logistic growth equation. The absolute growth rate (AGR) was calculated and is shown in the right panel. The error bars represent SD (n = 3). (A)∼(C). Tolerance to a high culture temperature. (D)∼(F). Tolerance to ethanol inhibition. (G)∼(I). Tolerance to acetate inhibition. (J)∼(L). Tolerance to acidic pH.

To measure whether TSH1 is tolerant to high temperatures, we investigated its growth characteristics by culturing TSH1 at higher temperatures. The growth curve analysis showed that TSH1 exhibited only minor changes in maximum cell concentration and the time required to enter the logarithmic growth phase as the temperature increased from 30°C to 40°C (Figure 2A); in contrast, BY4743 exhibited obvious growth inhibition with rising temperatures (Figure 2B). Moreover, TSH1 displayed better growth than BY4743 when the culture temperature reached 35°C, suggesting that the optimum growth temperature of TSH1 is higher than that of BY4743. Consistent with the growth curve, the maximum absolute growth rate (AGR) of TSH1 was higher than that of BY4743 at each temperature point (Figure 2C). Whereas BY4743 required 9 to 10 hours to reach the stationary phase at 30°C (Figure 2B), which is the normal temperature used for yeast cultivation [36], [37], TSH1 only required approximately 5 to 6 hours (Figure 2A). Despite their close genetic relationship, this result suggests that TSH1 exhibits a much higher growth rate than BY4743. Taken together, these results demonstrate that TSH1 has a rapid growth rate and can keep the growth characteristics in a wide range of tolerance to temperatures from 30°C to 40°C, which is quite excellent performance among reported wild type *S. cerevisiae*, suggesting that TSH1 can better resist the temperature fluctuation in solid-state fermentation.

To measure whether TSH1 is tolerant to ethanol and acetate, we investigated its growth characteristics by culturing TSH1 in medium with different ethanol and acetate concentrations. Through an ethanol tolerance assay, we found that the maximum AGR of TSH1 is higher than that of BY4743 at 5% and 6.5% ethanol (Figure 2F). Moreover, an increase in ethanol concentration from 0 to 6.5% only reduced the maximum AGR of TSH1 by 32.5%, whereas a 56.6% reduction was observed for BY4743 (Figure 2F). These results showed that TSH1 has a higher ethanol tolerance compared with BY4743.

Furthermore, we found that TSH1 showed a marked tolerance to acetate. The growth of TSH1 was almost unaffected as the acetate concentration increased from 2 g/L to 4 g/L (which will cause significant inhibition to many wild-type yeasts reported [34], [35]) (Figure 2G), whereas significant inhibitory effects on the maximum cell concentration and time required to reach the stationary phase were detected for BY4743 (Figure 2H). Consistently, an AGR analysis showed that the maximum AGR values for TSH1 were significantly higher than those for BY4743 and were less influenced by the presence of acetate (Figure 2I). Collectively, these results demonstrated that TSH1 had a strong tolerance to product inhibition, and potential adapt well to the poor mass transfer conditions of solid-state fermentation.

As acidic bacteriostatic agents widely used for storage of sweet sorghum [38]–[41] that can cause lower pH of sweet sorghum stalks during long-term storage, we further investigated TSH1's tolerance to acidic pH.

In an acidic pH tolerance assay, we found that the TSH1 growth curve was not significantly altered when the pH value of the medium was decreased from 5.0 to 3.0 (Figure 2J). In contrast, BY4743 exhibited a significant decrease in the stationary phase cell concentration (up to 21.8%) and an extension of the time required to reach stationary phase (Figure 2K). AGR analysis further demonstrated that TSH1 grew faster than BY4743 at each pH level; the growth of TSH1 exceeded that of BY4743 by 33.3% when the pH was reduced to 3.0 (Figure 2L). The excellent acidic pH tolerance of TSH1 should allow the direct fermentation of sweet sorghum stored under acidic conditions, possibly eliminating the need for pretreatment steps.

### TSH1 exhibits excellent performance in solid-state fermentation

To further evaluate the capability of TSH1 in solid-state fermentation, we examined fermentation using 500-mL flasks filled with crushed sweet sorghum stalks as the substrate at different temperatures and moisture contents, which are the two key factors affecting solid-state fermentation.

**Figure 3:**
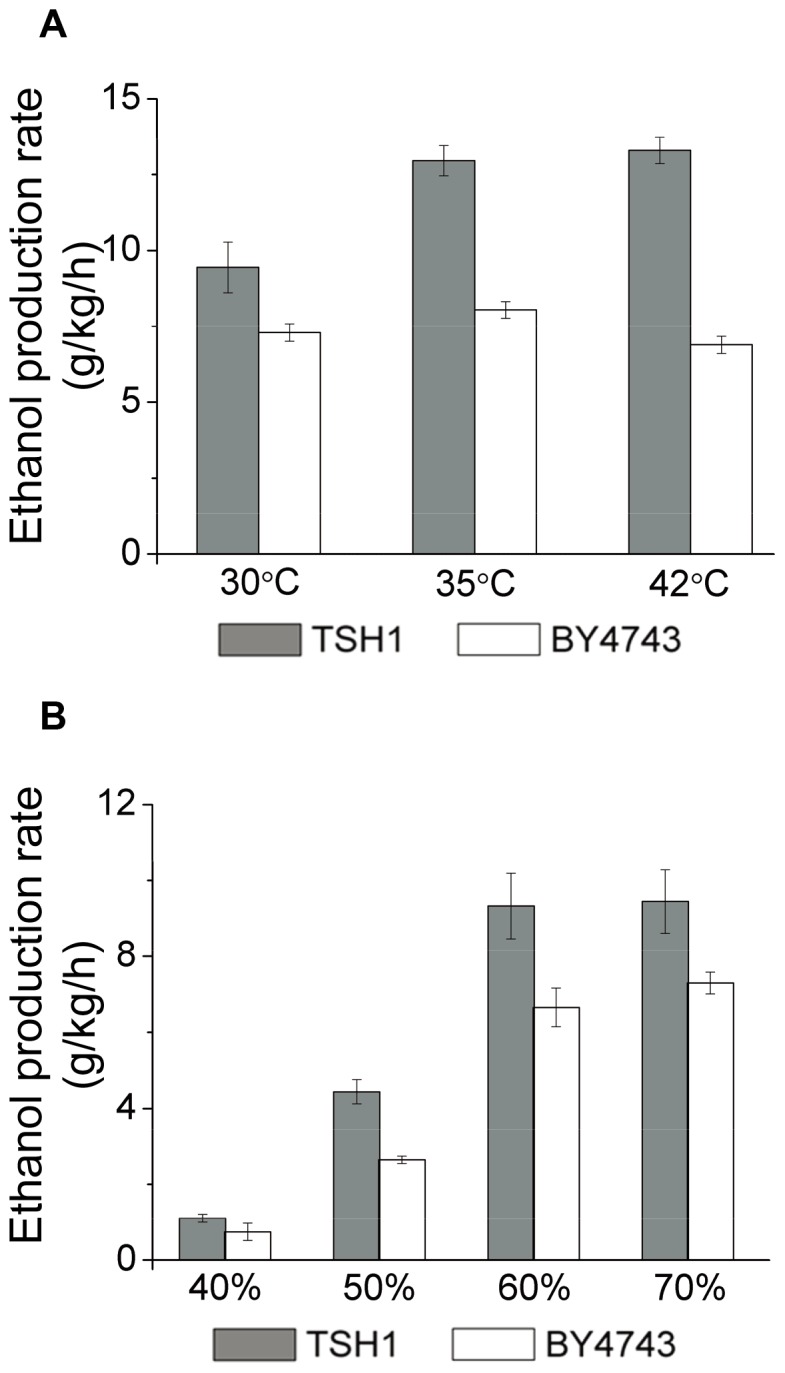
Temperature and moisture tolerance analysis in solid-state fermentation. (A). High temperature tolerance of TSH1. Solid-state fermentation with TSH1 was performed at the indicated temperatures for 12 hours using crushed sweet sorghum stalks as the substrate. BY4743 served as the control strain. The ethanol concentrations were measured by HPLC at the start and end points of fermentation. The ethanol production rate represents the ethanol weight produced per kg of substrate with 70% moisture content per hour. The error bars represent the SD (n = 3). (B). Low moisture content tolerance of TSH1. The crushed sweet sorghum stalks were pretreated to achieve the different moisture contents and subsequently loaded into the solid-state fermentation flasks. The error bars represent SD (n = 3).

We first conducted fermentation for 12 hours at different temperatures to test the tolerance of TSH1 to the local overheating that occurs in solid-state fermentation. As expected, the ethanol production rate of the control strain BY4743 increased slightly at 35°C compared with 30°C and notably decreased when the temperature was further raised to 42°C (Figure 3A). Conversely, TSH1 exhibited a significant increase in the ethanol production rate from 9.4 g/kg/h to 13.0 g/kg/h when the fermentation temperature was increased from 30°C to 35°C. Moreover, rather than decreasing at 42°C, TSH1 retained its fermentation capability at a level that was similar to that achieved at 35°C. These results showed that TSH1 performed well in solid-state fermentation and had an excellent ethanol production capability at a high temperature.

Next, we used crushed sweet sorghum stalks with different moisture contents (40%, 50%, 60%, and 70%) to determine the effect of substrate moisture on TSH1 fermentation. The results (Figure 3B) showed that the ethanol production rate of both TSH1 and BY4743 remained roughly constant when the moisture content was above 60%; however, these rates decreased rapidly below 60% moisture content. Regardless, the ethanol production rate of TSH1 was much higher than that of BY4743 at each level of moisture content investigated; moreover, compared with TSH1, the negative effects on BY4743 were much more pronounced when the moisture content was lower than 60%. These results demonstrate that TSH1 is better able to produce ethanol from substrates with lower moisture contents.

### TSH1 exhibited stability in the industrial scale-up process

**Figure 4:**
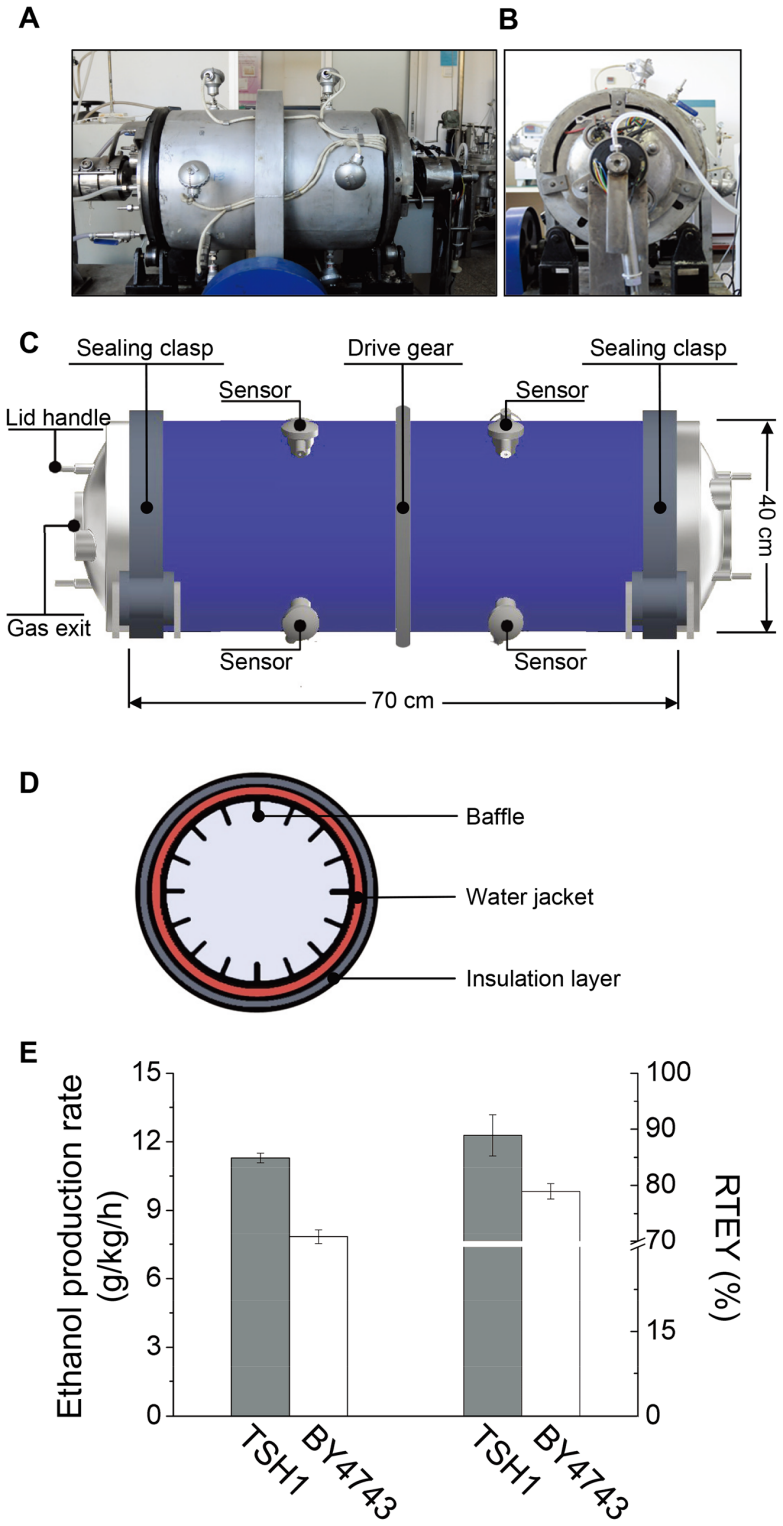
The performance of TSH1 in the 50-L rotary-drum fermenter. (A). Photograph of the 50-L rotary-drum fermenter. (B). Photograph of the lid of the 50-L rotary-drum fermenter. (C). Structure model of the 50-L rotary-drum fermenter. The fermenter consists of two lids and a tank. The tank and two lids are sealed with two sealing clasps, and handles are set on both lids; only one gas exit is fixed on the left side of the lid. Eight temperature sensors are evenly positioned on the tank to monitor the temperature at different positions, and a drive gear is fixed in the middle of the tank for rotation. (D). Structure model of the fermenter (crosscut view). The fermenter is covered with an insulation layer for heat preservation and a water jacket for temperature regulation. Many baffles are fixed on the inside wall to enhance material and heat transfer. (E). Solid-state fermentation analysis of TSH1 in the 50-L fermenter. Crushed sweet sorghum stalks (14 kg) inoculated with TSH1 were loaded and fermented at 30°C with a 0.5 rpm rotary speed for 12 hours. BY4743 served as the control strain. The ethanol concentrations were measured by HPLC at the start and end points of fermentation. The RTEY is defined as the ratio of ethanol weight produced compared to the theoretical yield based on sugar consumed (%). The error bars represent the SD (n = 3).

Having demonstrated that TSH1 was quite suitable for sweet sorghum solid-state fermentation at the flask scale, we further test whether TSH1 solid-state fermentation can be scaled up to the industrial level. As a first step toward scaling up, we designed a 50-L rotary-drum fermenter (Figure 4C, 4D), taking into consideration the stress tolerance and superior fermentation characteristics of TSH1 (Figure 4A, 4B).

The loading capacity of the 50-L fermenter is 14 kg of crushed sweet sorghum stalks; when mixed with the fermentation strain, each batch had a loading coefficient of approximately 55% (v/v). After 12 hours of fermentation at 30°C with a rotary speed of 0.5 rpm, we found that the average ethanol production rate of TSH1 was approximately 11.3 g/kg/h (with an RTEY of approximately 92.6%), whereas an average ethanol production rate of only 7.8 g/kg/h (with an RTEY of 78.9%) was achieved for BY4743 (Figure 4E). These results showed that TSH1 had an excellent ethanol production rate and RTEY in large-scale solid-state fermentation. The ethanol production rate of TSH1 after scale-up was much higher than that achieved using flasks (9.4 g/kg/h), indicating that more efficient fermentation was achieved using the rotary-drum fermenter.

To further scale up the fermentation, we next designed and built three rotary-drum fermenters with step-up pilot scales that were designed based on the 50-L fermenter with volumes of 5-m^3^, 40-m^3^, and 127-m^3^. Consistent with the 50-L fermenter, we found that both the average ethanol production rate (11.1±0.39 g/kg/h) and RTEY (88±0.8%) were quite stable, regardless of variations in the fermenter volume from 5-m^3^ to 127-m^3^ (Table 2). These results confirmed that TSH1 had an excellent ethanol production capacity in from sweet sorghum and exhibited fermentation stability in step-up rotary-drum fermenters. Additionally, these results suggested that the rotary-drum fermenter design took advantage of the characteristics of TSH1 and maximized its production efficiency.

**Table 2:** Ethanol rate and RTEY of step-up enlarged fermenters.

Scale (m^3^)	Ethanol production rate (g/kg/h)	RTEY (%)
5	11.6±0.52	88.8±1.29
40	11.2±0.87	88.0±3.47
127	10.7±0.80	87.7±1.45
550	10.5±0.94	88.6±0.72

### Batch fermentation by TSH1 in a 550-m^3^ fermenter

**Figure 5:**
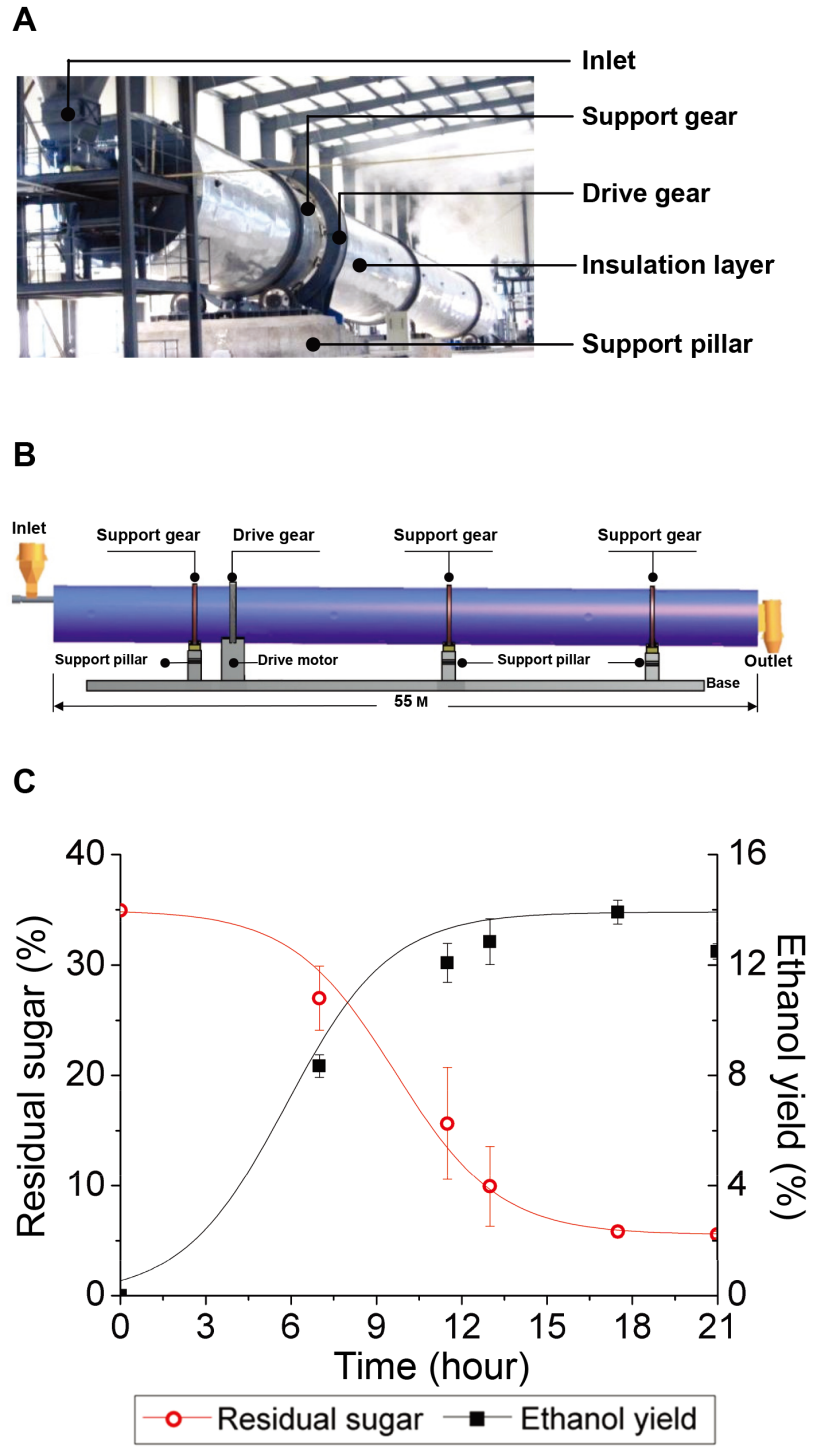
Fermentation of TSH1 in the 550-m3 fermenter. (A). Image of the 550-m^3^ fermenter in the workshop. (B). Structure model of the fermenter. The fermenter is fixed on a base supported by three support gears and rotates via a drive gear; a feed inlet and discharge outlet can cross the fermenter on conveyors. A 5-degree angle between the tank and base is applied to enhance substrate transfer. The surface of the fermenter is covered with a layer of insulation for heat preservation. (C). Residual sugar and ethanol yield during fermentation. Crushed sweet sorghum stalks (96 tons) were fermented by TSH1 at 30°C for 21 hours, and samples were collected at the indicated times. The residual sugar was measured by the DNS method and is represented as a percentage (by weight) of the total sugar (including sucrose, glucose, and fructose) that remained unfermented in 1 g of substrate. The ethanol yield was measured by HPLC and is represented as the percentage (by weight) produced by 1 g of substrate. The error bars represent the SD (n = 3).

Given that the 127-m^3^ fermenter was still not large enough to be cost-effective for utilizing sweet sorghum in industrial-scale solid-state fermentation, we further designed and constructed a commercial demonstration system with a 550-m^3^ rotary-drum fermenter (Figure 5A, 5B).

For each batch, we loaded approximately 96 tons of crushed sweet sorghum stalks inoculated with TSH1 into the fermenter using the conveyors, and we tracked the fermentation up to 21 hours. Figure 5C shows that a period of only 15 hours was required for the ethanol accumulation to reach the highest point, as the sugar was exhausted during this period. The ethanol production rate analysis further showed that the average ethanol production rate for the first 12 hours can reach 10.5 g/kg/h and that RTEY can reach approximately 88% (Table 2), which is similar to that achieved with the 127-m^3^ fermenter, suggesting a successful scale-up process. These results showed that the fermentation system developed in this study has a short fermentation period and high ethanol production capability, demonstrating that the solid-state fermentation method based on a rotary-drum fermenter with TSH1 as the fermentation strain exhibited excellent performance and can be applied to the industrial process.

An economic analysis showed that the energy input:output ratio was approximately 1∶2.6, as shown in Table 3, and that the ethanol cost per ton was approximately US $740.08, as shown in Table 4. These costs are reduced compared with those of corn-based fuel ethanol and sugarcane-based fuel ethanol [1], [2], [42], [43]. These results collectively demonstrated that the solid-state fermentation system can significantly reduce the sweet sorghum ethanol production cost and achieve a highly efficient and low-cost solution to non-food bioethanol production.

**Table 3:** Energy input and output based on 550-m3 fermenter data.

Item		Input (kcal×1000)	Item		Output (kcal×1000)
Electricity		284.5	Ethanol		7024
Steam		4098	Vinasse		4376
	Total	4382.5		Total	11400

**Table 4:** Economic analyses per ton of ethanol based on 550-m3 fermenter.

Item	Unit Price (USD)	Total amount	Cost (USD/ton)
Feedstock			
Sweet sorghum a	30	16 tons	30×16 = 480
Transport b	2.13	16 tons	2.13×16 = 34.08
Storage fee c	5	16 tons	5×16 = 90
Utility d			
Electricity	0.1	332 kw·h	0.1×332 = 33.2
Steam	8	2.5	20
Water	1	4 tons	1×3 = 4
Yeast + Enzymes d			26.5
Labor d			50
Maintenance d			20
Depreciation d			64.3
Management Fees d			18
Finance d			30
Bagasse d	20	6 tons	−20×6 = −120
Total			740.08

a Average price of sweet sorghum was around US $ 30/t as reported [52].

b Biomass loaded fee is US $ 1.10/t and then US $ 0.103/t/km transported [51], therefore, the transport cost of our plant is around US $ 2.13/t (the maximum feedstock collection radius is 10 km).

c Using SO_2_ for storage, the cost is around US $ 5/t.

d The capital cost and other fees were the same as reported [52].

## Discussion

Bioethanol based on non-food crops, particularly sweet sorghum, is currently attracting global attention. Compared to liquid-state fermentation utilizing sweet sorghum juice obtained by pressing or other types of fermentation, solid-state fermentation has certain advantages, including increased sugar utilization, lower capital cost, and reduced wastewater output [12], [14], [15]. However, breakthrough progress in the implementation of sweet sorghum solid-state fermentation for industrial-scale ethanol production has not been achieved for some time because of two major technology bottlenecks: suitable fermentation strains and efficient fermenter design.

To find a suitable fermentation strain, we screened and identified the *S. cerevisiae* strain TSH1 from soil in which sweet sorghum stalks were stored (Figure 1). Growth characteristic analysis showed that TSH1 had excellent growth adaptability, demonstrating tolerance to product inhibition, acidic pH, and a wide range of temperatures. TSH1 also was a strong performer in fermentation at high temperatures and low moisture contents (Figure 2, 3).

Different from many industrial *S. cerevisiae* strains that are not genetically modified [37], [44]–[46], when cultured at 40°C, TSH1 retains its rapid growth and good production. In fact, these traits are retained even at 42°C (Figure 3), suggesting that TSH1 can accommodate itself well to the poor heat transfer environment in solid-state fermentation.

The ethanol and acetate tolerance of TSH1 might be derived from evolutionary selection pressure under its specific living conditions. We noted that the ethanol and acetate tolerance of TSH1 was not as high when compared with some reported *S. cerevisiae* strains [37], [45]–[47], which might indicate the potential for further strain engineering for production enhancement.

Previous studies have shown that sulfur dioxide is the most effective acidic bacteriostatic agent for the long-term storage of sweet sorghum [38], [41], [48], [49]. In our 127-m^3^ and 550-m^3^ scale production studies, we found that the strong acidic pH tolerance of TSH1 allowed the direct use of sweet sorghum stalks treated with sulfur dioxide without the need for any pretreatment to adjust the pH value. This significantly simplified the solid-state fermentation procedure and also reduced costs at the industrial scale.

During the progressive scale-up of fermenters from 50-L to 550-m^3^, we found that TSH1 achieved an ethanol production rate of 11.1±0.39 g/kg/h and an RTEY of 88±0.8% (Figure 4, Table 2). These data exceed the previously reported values for industrial solid-state fermentation of sweet sorghum [10]–[12], [18], further confirming the feasibility and capability of TSH1 for solid-state fermentation.

With regard to the second bottleneck, we developed rotary-drum fermenters to improve heat and mass transfer via the addition of baffles with different orientations and increasing the slope angle between the fermenter and base (Figure 4, 5). After optimizing the baffle distribution and fermenter rotary speed, the fermentation of up to 96 tons of crushed sweet sorghum stalks could be completed by batch fermentation in the 550-m^3^ rotary-drum fermenter in only approximately 20 hours, with an 88% RTEY (Table 2). These results showed that the biggest industrial-scale sweet sorghum solid-state fermentation system had been successfully established in the world, demonstrating the suitability of the newly designed rotary-drum fermenter for the large-scale solid-state fermentation of sweet sorghum.

We also evaluated the market competitiveness of the 550-m^3^ rotary-drum fermentation system: the system achieved an energy input:output ratio of approximately 1∶2.6 for the production of one ton of ethanol as the by-product vinasse can also be utilized (Table 3). Our economic analysis showed that the ethanol cost per ton was approximately US $740.08 (Table 4) for batch fermentation, which had significant market competitiveness compared to ethanol produced from corn and cassava in China and other techniques reported for production of sweet sorghum ethanol [1], [2], [43], [50], [51]. Taken together, these data suggested that the solid-state fermentation platform is very cost effective and competitive for the bioethanol market.

## Conclusion

Industrial-scale bioethanol production using sweet sorghum is limited by two major technology bottlenecks: suitable fermentation strains and efficient fermenter design. In the present study, we screened and identified a novel *S. cerevisiae* TSH1 strain which had excellent ethanol fermentative capacity and ability to withstand stressful solid-state fermentation conditions. Next, we constructed the rotary-drum fermenters from 50-L to 550-m^3^, exhibiting the feasibility and high-efficiency in using TSH1 as fermentative strain to set up solid-state fermentation of ethanol from sweet sorghum stalks in industrial scale. An economic analysis further demonstrated that both the energy input:output ratio and the production cost are very market competitive using the 550-m^3^ fermentation system. Therefore, this fermentation system can contribute to the development of non-food bioethanol production, significantly reducing the cost of utilizing sweet sorghum for ethanol production.
